# Interdependency and differential expression of ERK1 and ERK2 in breast and melanoma cell lines

**DOI:** 10.1186/s43046-024-00233-3

**Published:** 2024-09-16

**Authors:** Shuvojit Moulik, Sayantani Karmakar, Asmita Basu, Mahammad Ali, Amitava Chatterjee

**Affiliations:** 1 Research and Development Wing, Suraksha Diagnostics Pvt. Ltd., Newtown, Kolkata, West Bengal India; 2 CWF Labs: Transdisciplinary Healthcare & Research, Bolpur, West Bengal India; 3 Department of Analytical Chemistry, Jadavpur University, Kolkata, West Bengal India; 4 Chittaranjan National Cancer Institute (CNCI), Kolkata, West Bengal India

**Keywords:** Melanoma, Breast cancer, ERK, Matrix metalloprotease

## Abstract

**Background:**

Regulatory mechanism of ERK1 and ERK2, their mechanisms of action, and how they impact on development, growth, and homeostasis of different organisms have been given much emphasis for long. ERK1 and 2 though are isoforms of ERK mitogen-activated protein kinase but are coded by two different genes MAPK3 and MAPK1 respectively and show differential expressions and interdependency in different cancer cell lines. Our previous investigations substantially stated the effect of ERK1 and ERK2 on different extracellular molecules like MMPs and integrins, responsible for cell growth and differentiation. Here, we aim to study individual roles of ERK1 and ERK2 and their interdependency in progression and invasiveness in various cancer cell lines.

**Methods:**

Different cancer cell lines namely B16F10 (melanoma), MCF7, and MDAMB231 (breast cancer) for studying this particular question were used. Methodologies like gelatin zymography, immunoprecipitation, Western blotting, cell invasion assay, wound healing assay, siRNA transfection, and double transfection procedures were followed for our study.

**Results:**

Our findings suggest compensation for ERK2 deficiency by pERK1, clear ERK2 predominance in MCF7 cell line, ERK1-ERK2 interdependency in MDAMB231 cells with regard to compensating each other, and significant role of both ERK1 and ERK2 in modulation of MMP9.

**Conclusion:**

If summarized, our results prove the contribution of ERK2 in compensating ERK1 loss and vice versa and an evident role of ERK1 in cancer cell invasiveness.

## Background

The complexity of the much-anticipated linear mitogen-activated protein kinase (MAPK) pathway stands true still as, after number of well-directed small molecule-based inhibitor failed to create a positive effect in cancer treatment and prognosis. Extracellular signal-regulated kinase (ERK) being one of the most significant members of this pathway add to this complexity with its differential expression of otherwise identical isoforms ERK1 and ERK2 [1, 2]. There is hardly much structural difference with 84% similarity in amino acid levels between the two isoforms [3–6]. In ERK2, the peptide binding site is blocked by Tyr 185, one of the two residues that are phosphorylated in the active enzyme. Even for substrate phosphorylation, ERK1 and ERK2 have a very similar specificity [7, 8]. In contrary to the analogous structural and functional attributes of the two isoforms, there are recent evidences, suggesting qualitative and quantitative differences between ERK1 and ERK2 that can exert differential effect on several cellular functions including cell differentiation and cell proliferation [9–11]. Cell differentiation and cell proliferation are two of the pivotal forces for cancer progression and invasiveness [12, 13]. In our previous investigations, we explained the considerable effect of ERK1 and ERK2 on various extracellular molecules like matrix metalloproteinases (MMP2, MMP9) and integrins, which are majorly responsible for cancer cell progression and tumor invasiveness [14, 15]. In this particular study, we aim to find out the distinguishable role of ERK1 and ERK2 and their interdependency, if any with respect to cancer cell progression and invasiveness. Three different cell lines B16F10 (melanoma), MCF7, and MDAMB231 (breast cancer) were used in the model system for this specific study.

## Materials and methods

### Cell culture

Human breast cancer cell lines MCF-7 and MDA-MB-231 and murine melanoma cancer cell line B16F10 were obtained from NCCS, Pune, and were grown and maintained in required medium-like Minimal Essential Medium (GIBCO), Dulbecco’s Modified Eagle’s Medium (GIBCO), and McCoys’ 5A Medium (GIBCO) containing 10% fetal bovine serum (LifeTech, BioWhittaker) in a CO_2_ incubator at 37 °C.

#### Zymography

For gelatin zymography, gelatin (Sigma), Triton-X (Promega), NaCl, CaCl_2_, gelatin-Sepharose 4B beads (Amersham) were used. The method was followed according to the protocol by Moulik et al. [14].

#### Immunoprecipitation and Western blotting

Protein extraction from cells and immunoprecipitation assay was done following the protocol of Moulik et al. [14]. The protein content was estimated by Lowry’s method. Equal amount of protein was taken and immunoprecipitated from the supernatant using required primary antibodies and protein-G agarose beads and shaking them overnight at 4 °C. The resultant immune complex was thoroughly washed thrice in PBS and then subjected to Western blotting.

For Western blotting, the materials used were as follows: acrylamide (Promega), bis-acrylamide (Sigma), Tris (Promega), sodium dodecyl sulfate (Biogene), ammonium persulfate (LifeTech), glycine and stain (Coomassie Brilliant Blue), bromophenol blue, methanol, acetic acid, and TEMED for SDS page. Primary antibody used was anti-phospho-ERK, anti-ERK, anti-MMP2, anti-MMP9, anti-JNK, anti-phospho-JNK, anti-PI-3 K, anti-STAT-3 anti-integrins antibody, or as required (Sigma, Promega, Santa Cruz) and 2nd antibody both monoclonal and polyclonal (Promega/Santa Cruz) and substrate used was NBT/BCIP or Femto substrate for ECL (Pierce) NaCl, Tris, Tween-20, Tris, glycine, methanol, BSA, and nitrocellulose membrane. The method was followed according to Moulik et al. [14].

### Cell invasion assay

For the assay, Millicell inserts (Millipore) and Matrigel (BD Biosciences) were used. The protocol was followed according to Moulik et al. [14]. Briefly, 24-well transwell plate (Corning) with 12 inserts were taken, and the lower chamber of each well was poured with 600-ml MEM SFCM. Control and fibronectin (Fn)-treated cells (100,000 cells/insert) were seeded in triplicate on membrane in the upper chamber of the insert. Cells were then allowed to grow for 24 and 48 h. After 24 and 48 h of incubation, media was pipetted out from membrane. SFCMs from lower chambers were collected and centrifuged at 3000 r.p.m for 3 min. The membranes of the inserts were washed thrice with PBS. Cells were then fixed with 4% formaldehyde solution, followed by washing with PBS. Cells were then stained with Gill’s hematoxylin for 10 min. Membranes were then washed thoroughly in running water. The upper side of the membranes was scraped with buds; membranes were then cut and mounted with glycerol. The cells migrated through the membrane pore were observed under microscope.

### Wound healing assay

Cells were grown as a monolayer on culture plates in the absence (C) and in presence of 50 µg/ml theaflavin at 37 °C for 24 h (E). The monolayer was scratched with a sterile pipette tip, followed by washing with serum-free complete medium (SFCM) to remove cellular debris. Cell migration across the wound was observed by microscope and documented by photographs at 0 h, 6 h, 24 h, and 48 h [14].

### siRNA transfection

#### Single transfection procedure

Cells were plated 24 h prior to transfection. The cells were incubated at 37 °C for 24 h under 5% CO_2_. After incubation, the plate was washed with PBS (once) and layer with 1.75-ml Opti-MEM. The following mix was prepared and incubated at room temperature for 5 min.

- Tube 1: siRNA: 100 nM (stock conc.: is 100 µM, siRNA: 2 µl) and Opti-MEM: 198 µl
- Tube 2: Oligofectamine: 4 µl and Opti-MEM: 46 µl

The content of both the tubes was mixed and incubated for further 30 min. siRNA-lipid mix on the cells (dropwise with constant swirling) was layered. The plate was incubated at 37 °C for 4 h. Fresh media was added after 4-h incubation and incubate further 24 h. Cells were analyzed depending upon the experiment.

*Double transfection (combinatorial transfection) procedure* involves two rounds of siRNA transfection, and second round of transfection was done in the similar manner as described above after 24 h of the first round. Scramble control for siRNA was similarly prepared.

### Constitution of siRNA

1 × nuclease-free siRNA buffer was prepared from 5 × provided by Dharmacon (use nuclease-free water, also provided by Santa Cruz). For 100-nM siRNA, 1-ml 1 × nuclease-free siRNA buffer was used. Aliquots the siRNA were stored at − 20 °C.

## Results

**Fig. 1:**
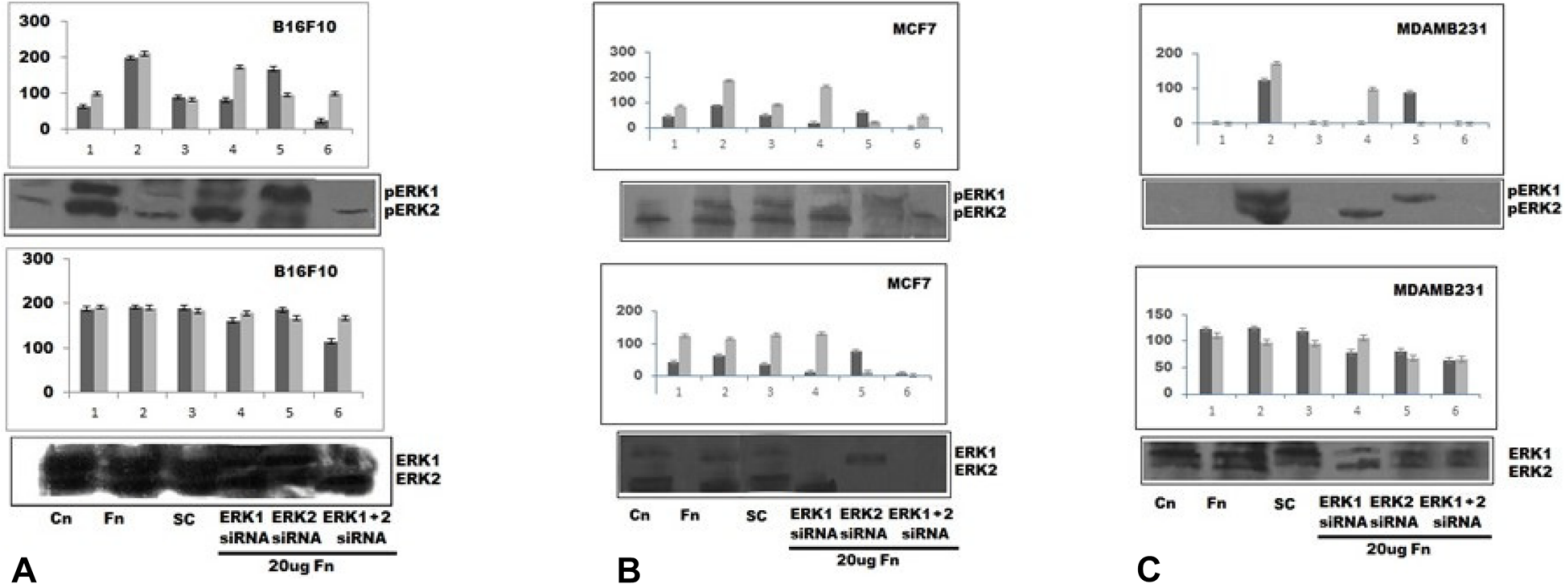
Effect of siRNA on activity and expression of ERK 1 and ERK 2 by immunoblot. For the transfection process, ERK1 siRNA, ERK 2 siRNA, ERK 1 + ERK 2 siRNA (sequential double transfection procedure), and negative control siRNA were transfected using Lipofectamine™ 2000 following standard protocol. Cells were then treated with 20-µg fibronectin (B16F10: 16 h, MCF7: 2 h, MDAMB231: 8 h), and the respective serum-free medium was subjected to Western blot by standard protocol. The immunoblot was probed for anti-pERK 1/2 and anti-ERK1/2 antibody. The blots representing B16F10 (**A**), MCF7 (**B**), and MDAMB231 (**C**) cells were developed using respective horse redox peroxidase (HRP) coupled second antibodies. The color was developed using West Femto as substrate. *β*-tubulin was used as loading control (figure not shown). The accompanying graph represents the comparative densitometric/quantitative analysis of the band intensities using ImageJ launcher (version 1.4.3.67) and arranged in the similar order as of the lanes. Data are means ± SEM of three experiments

Figure 1 shows the effect of ERK1 and ERK2 siRNA on the activity and expression of ERK1 and ERK2. In Fig. 1A, the activity of pERK1 increases considerably in Fn-treated B16F10 cells, which gets lowered by almost 50% when ERK1 siRNA is administered. Following administration of ERK2 siRNA, the level of ERK1 again increases considerably. The sequential double transfection of ERK1 + ERK2 siRNA resulted in almost complete depletion of pERK1. The expression of total ERK1 was more or less equal throughout all the lanes except during the double transfection of ERK1 + ERK2 siRNA where there was around 30% reduction in levels of ERK1 with respect to control. Figure 1A also shows the activity and expression of ERK2. The levels of pERK2 increased with Fn, negligibly altered with ERK1 siRNA, and depleted noticeably with the double transfection of ERK1 and ERK2 siRNA. The total protein levels of ERK2 remained almost similar throughout. The scramble control (SC) did not show any difference with respect to control. Figure 1B shows the levels of pERK1/pERK2/ERK1/ERK2 in MCF7 cells. pERK1 increased subsequently with 20-µg Fn and decreased in the presence of ERK1 siRNA. The activity level of ERK1 increased slightly with respect to Fn-treated cells when ERK2 siRNA was administered and the levels reduced to negligible amounts when ERK1 + ERK2 siRNA was doubly transfected. The total protein levels of ERK1 remained unaltered until ERK1 siRNA was introduced singly or in the double transfection. The activity levels of pERK2 increased in Fn-treated cells and only went down when ERK2 siRNA was transfected singly or in the combinatorial transfection. The levels of activity and expression of ERK1 and ERK2 in MDAMB231 cells are portrayed in Fig. 1C. There was a considerable increase of pERK1 with Fn-treated cells with respect to control or scramble control, and the levels decreased with ERK1 siRNA treatment. pERK1 band intensifies with introduction of ERK2 siRNA but depleted when the combinatorial transfection (ERK1 + ERK2 siRNA) was introduced. The levels of total protein of ERK1 remained similar and got reduced when ERK1 siRNA was treated singly or via double transfection. The activity of pERK2 increased with Fn-treated MDAMB231 cells, remained higher in ERK1 siRNA-treated cells, and decreased to minimal levels when ERK2 siRNA was introduced singly or in combination with ERK1 siRNA. The expression of ERK2 remained similar all through and got reduced by around 20% with respect to control when ERK2 siRNA was treated singly or in combination with ERK1 siRNA. The scramble control (SC) did not show any difference with respect to control.

**Fig. 2:**
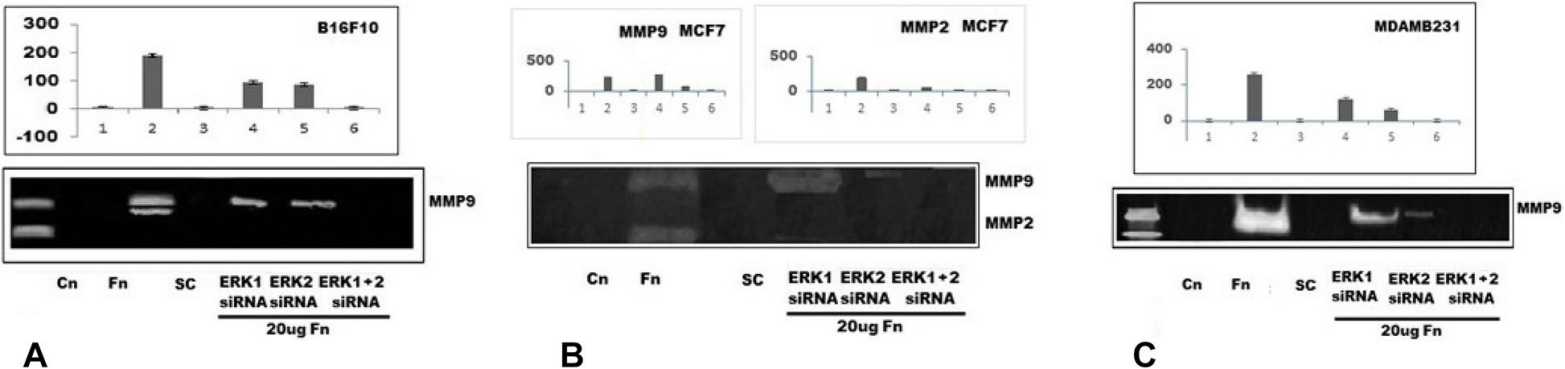
Effect of ERK1 and ERK2 siRNA on MMP2 and MMP9. For the transfection process, ERK1 siRNA, ERK2 siRNA, ERK1 + ERK2 siRNA (sequential double transfection procedure), and negative control siRNA were transfected using Lipofectamine.™ 2000 following standard protocol. Cells were then treated with 20-µg fibronectin (B16F10: 16 h, MCF7: 2 h, MDAMB231: 8 h), and the respective serum-free medium was subjected to zymography by standard protocol. The zymograms representing B16F10 (**A**), MCF7 (**B**), and MDAMB231 (**C**) cells were developed following standard incubation procedures. The accompanying graph represents the comparative densitometric/quantitative analysis of the band intensities using ImageJ launcher (version 1.4.3.67) and arranged in the similar order as of the lanes. Data are means ± SEM of three experiments. Each zymogram is accompanied with marker (leftmost lane) projecting the exact size of MMP2 or MMP9

Figure 2 represents MMP 2 and MMP 9 activity against ERK1 and ERK2 siRNA. In B16F10 cells (Fig. 2A), MMP 9 levels increased in Fn-treated cells with respect to control. MMP 9 levels reduced considerably when ERK 1 and ERK 2 siRNA was introduced separately but depleted to undetectable levels when double transfection with ERK1 and ERK2 siRNA was done. Pro MMP9 was noticed in Fn-treated cells but could not be traced in other lanes. MCF7 cells (Fig. 2B) secreted both MM9 and MMP2 when cells were treated with 20-µg fibronectin. When ERK1 siRNA was introduced, the levels of MM9 remained unaltered, but MMP2 was undetectable. With ERK2 siRNA, the activity of MMP9 became negligible, while MMP2 was untraceable. When combinatorial transfection with ERK1 and ERK2 siRNA was done, both the levels MMP2 and MMP9 were beyond detection limit. In Fig. 2C, MDAMB231 cells released very high levels of MMP9 in Fn-treated cells, which got lowered by appreciable amounts in ERK1 siRNA-treated cells. It went down even lower with ERK2 siRNA treatment and dropped to insignificant amount with sequential double transfection of ERK1 and ERK2 siRNA.

**Fig. 3:**
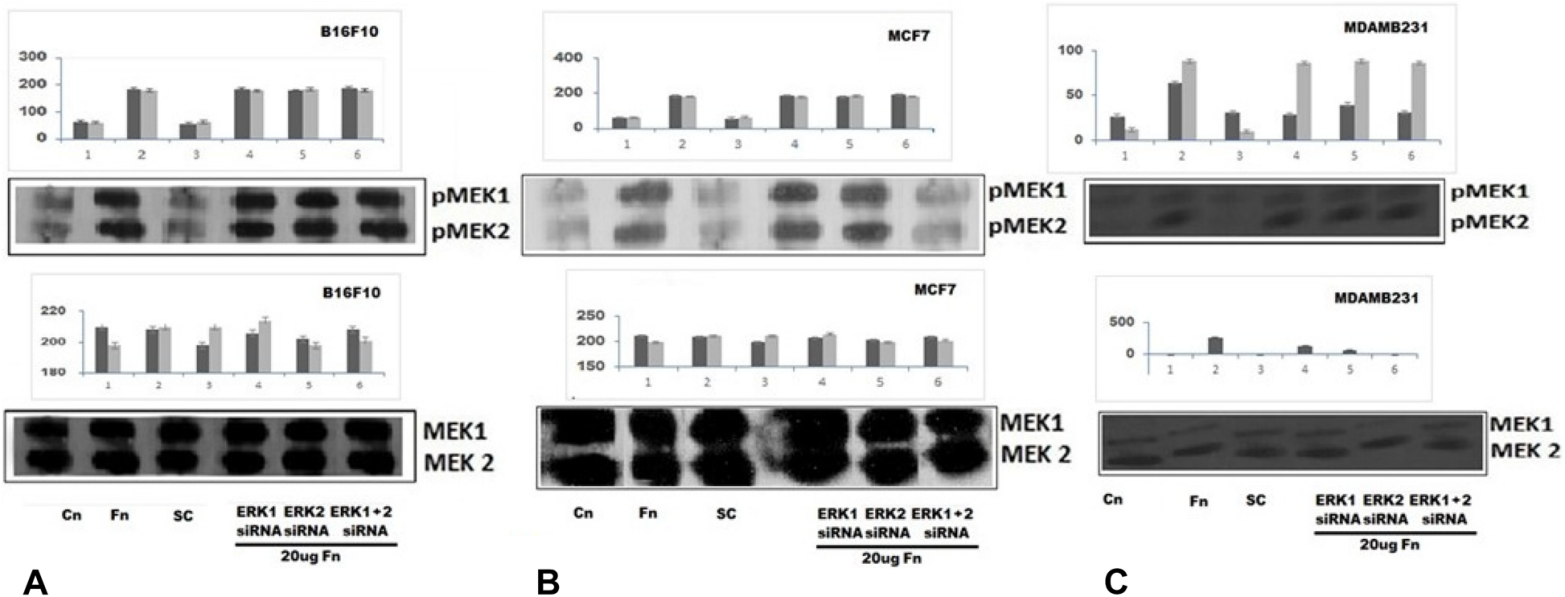
Effect of ERK1 and ERK2 siRNA on activity and expression of MEK1/2. For the transfection process, ERK1 siRNA, ERK2 siRNA, ERK1 + ERK2 siRNA (sequential double transfection procedure), and negative control siRNA were transfected using Lipofectamine™ 2000 following standard protocol. Cells were then treated with 20 µg fibronectin (B16F10: 16 h, MCF7: 2 h, MDAMB231: 8 h), and the respective serum-free medium was subjected to Western blot by standard protocol. The immunoblot was probed for anti-pMEK1/2 and anti-MEK1/2 antibody. The blots representing B16F10 (**A**), MCF7 (**B**), and MDAMB231 (**C**) cells were developed using respective horse redox peroxidase (HRP) coupled second antibodies. The color was developed using West Femto as substrate. *β*-tubulin was used as loading control. The accompanying graph represents the comparative densitometric/quantitative analysis of the band intensities using ImageJ launcher (version 1.4.3.67) and arranged in the similar order as of the lanes. Data are means ± SEM of three experiments

Figure 3 shows the effect of ERK modulation on the levels of MEK activity and expression. The expression of pMEK1/2 in B16F10 cells (Fig. 3A) became higher with Fn treatment and remained so during all types of siRNA transfection. The total protein levels of MEK1/2 did not vary considerably throughout Fn-treated and -untreated cells. In Fig. 3B, pMEK1/2 went higher with Fn treatment in MCF7 cells. The levels remained intact when ERK1 and ERK2 siRNA was introduced separately but reduced to minimal amount when ERK1 and ERK2 siRNA was introduced in combination. The expression of MEK1/2 in dephospho form was constant throughout. In Fig. 3C (MDAMB231 cells), the activity of MEK1/2 went higher with Fn treatment and did not change appreciably with siRNA transfections. The total protein levels of MEK1/2 also went higher with Fn treatment but otherwise remained intact throughout.

**Fig. 4:**
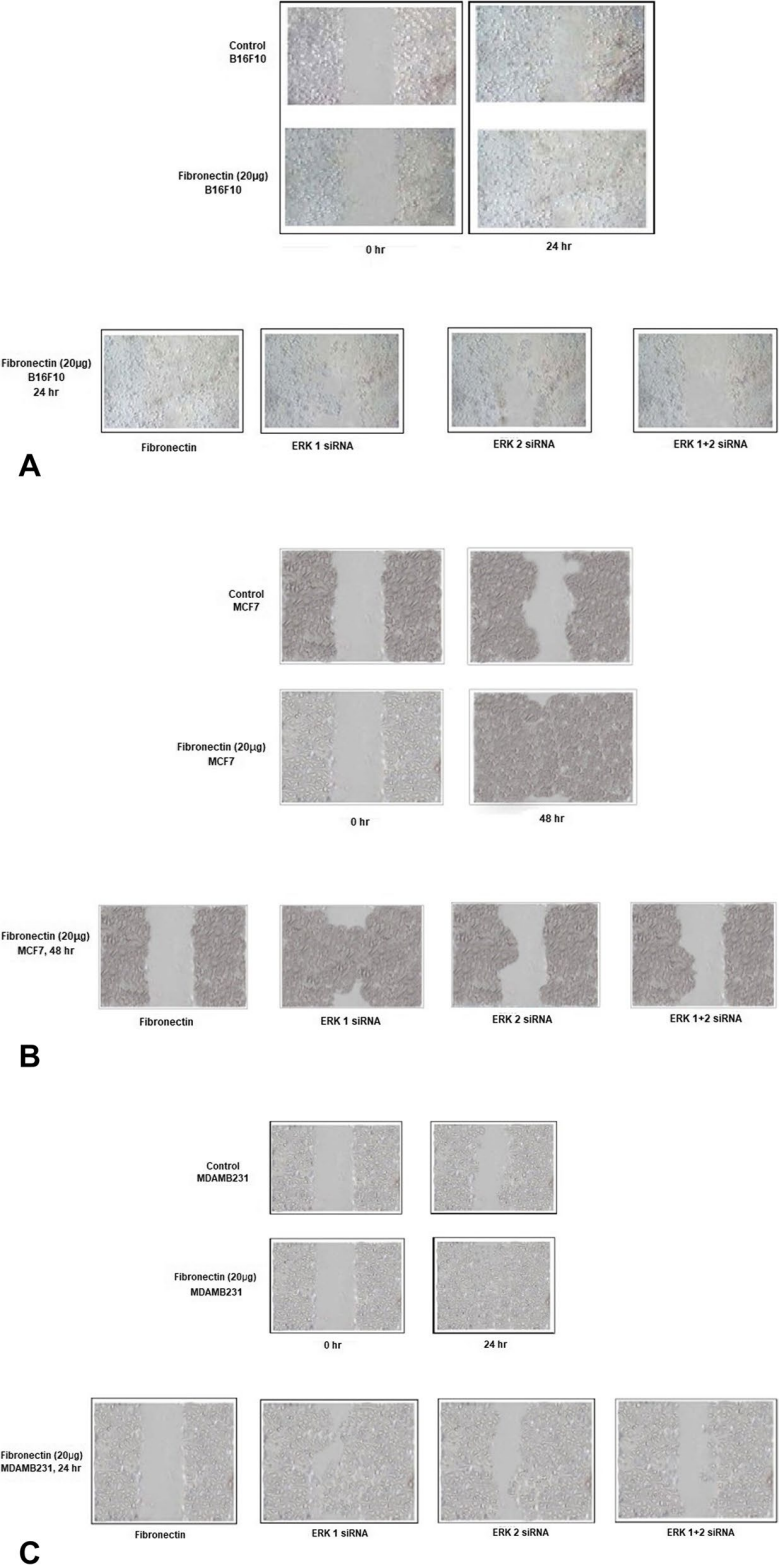
Effect of inhibition of ERK1 and ERK 2 on cell migration. **A** B16F10 cells (300,000 cells/ml) were grown in serum free culture medium in absence (control) and in presence of fibronectin (20 µg per/ml) for 16 h. The monolayer was scratched with a sterile pipette tip, followed by washing thrice with SFCM to remove cellular debris. The cells were maintained in fresh SFCM and cell migration was observed by microscopy and documented by photography at 0 h and 24 h. ERK1 siRNA, ERK 2 siRNA and combinatorial transfection of ERK1 + ERK2 siRNA treated cells were cultured in a monolayer in absence and presence of fibronectin (20 mg/ml) for 16 h. The monolayer was scratched with a sterile pipette tip, followed by washing thrice with SFCM to remove cellular debris. The cells were maintained in fresh SFCM and cell migration was observed by microscopy and documented by digital photography at 0 h and 24 h. **B** MCF7 cells (300,000 cells/ml) were grown in serum free culture medium in absence (control) and in presence of Fibronectin (20 µg per/ml) for 16 h. The monolayer was scratched with a sterile pipette tip, followed by washing thrice with SFCM to remove cellular debris. The cells were maintained in fresh SFCM and cell migration was observed by microscopy and documented by photography at 0 h and 24 h. ERK1 siRNA, ERK 2 siRNA and combinatorial transfection of ERK1 + ERK2 siRNA treated cells were cultured in a monolayer in absence and presence of Fibronectin (20 mg/ml) for 2 h. The monolayer was scratched with a sterile pipette tip, followed by washing thrice with SFCM to remove cellular debris. The cells were maintained in fresh SFCM and cell migration was observed by microscopy and documented by digital photography at 0 h and 48 h. **C** MDAMB231 cells (300,000 cells/ml) were grown in serum free culture medium in absence (control) and in presence of Fibronectin (20 µg per/ml) for 16 h. The monolayer was scratched with a sterile pipette tip, followed by washing thrice with SFCM to remove cellular debris. The cells were maintained in fresh SFCM and cell migration was observed by microscopy and documented by photography at 0 h and 24 h. ERK1 siRNA, ERK 2 siRNA and combinatorial transfection of ERK1 + ERK2 siRNA treated cells were cultured in a monolayer in absence and presence of Fibronectin (20 mg/ml) for 8 h. The monolayer was scratched with a sterile pipette tip, followed by washing thrice with SFCM to remove cellular debris. The cells were maintained in fresh SFCM and cell migration was observed by microscopy and documented by digital photography at 0 h and 24 h

Figure 4 portrays the effect of targeted inhibition of ERK1 and ERK2 on cell migration. B16F10 cells (Fig. 4A) showed an increased rate of cell migration with Fn treatment which dropped down completely when combination treatment of ERk1 and ERk2 siRNA was introduced. There was also an appreciable change in the rate of cell migration when ERK1 siRNA and ERK2 siRNA was treated separately. MCF7 cells (Fig. 4B) showed a higher rate of migration in cells with ERK1 siRNA treatment, with respect to control, ERK2 siRNA treatment, and with double transfection of ERK1 and ERK2 siRNA. In Fig. 4C, there was also an increased rate of cell migration with Fn-treated ERK1 siRNA-transfected MDAMB231 cells with respect to ERK2 siRNA transfection and the combinatorial transfection.

**Fig. 5:**
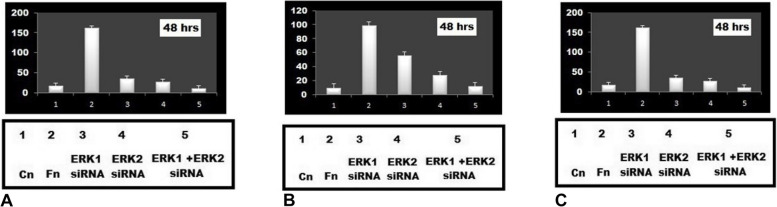
Effect of inhibition of ERK1 and ERK 2 on cell invasion. ERK1 siRNA, ERK2 siRNA, sequentially transfected ERK1 and ERK2 siRNA, and control siRNA-treated cells were cultured in transwell chambers in triplicate in the absence and presence of fibronectin (20 mg/ml) for 16 h. The chamber was inserted in DMEM containing 5% FBS as chemoattractant and grown for 48 h. The chambers were then removed and washed. The cells migrated on the membrane were observed under microscope. Number of cells migrated through transwell insert was counted per microscopic power field. **A**, **B**, and **C** represent the number of invaded B16F10, MCF7, and MDAMB231 cells, respectively

Figure 5 represents the invasion rate of cancer cells with respect to ERK1 and ERK2 siRNA treatments. In B16F10 cells (Fig. 5A), the invasion rate dropped noticeably with siRNA treatment (both separately and combination). The similar trend was found in MDAMB231 cells (Fig. 5C). In MCF7 cells (Fig. 5B), there was an increase rate of cell invasion with ERK1 siRNA treatment which gradually lowered down to basal levels with ERK2 siRNA treatment and with administration of double transfection of ERK1 and ERK2 siRNA.

**Fig. 6:**
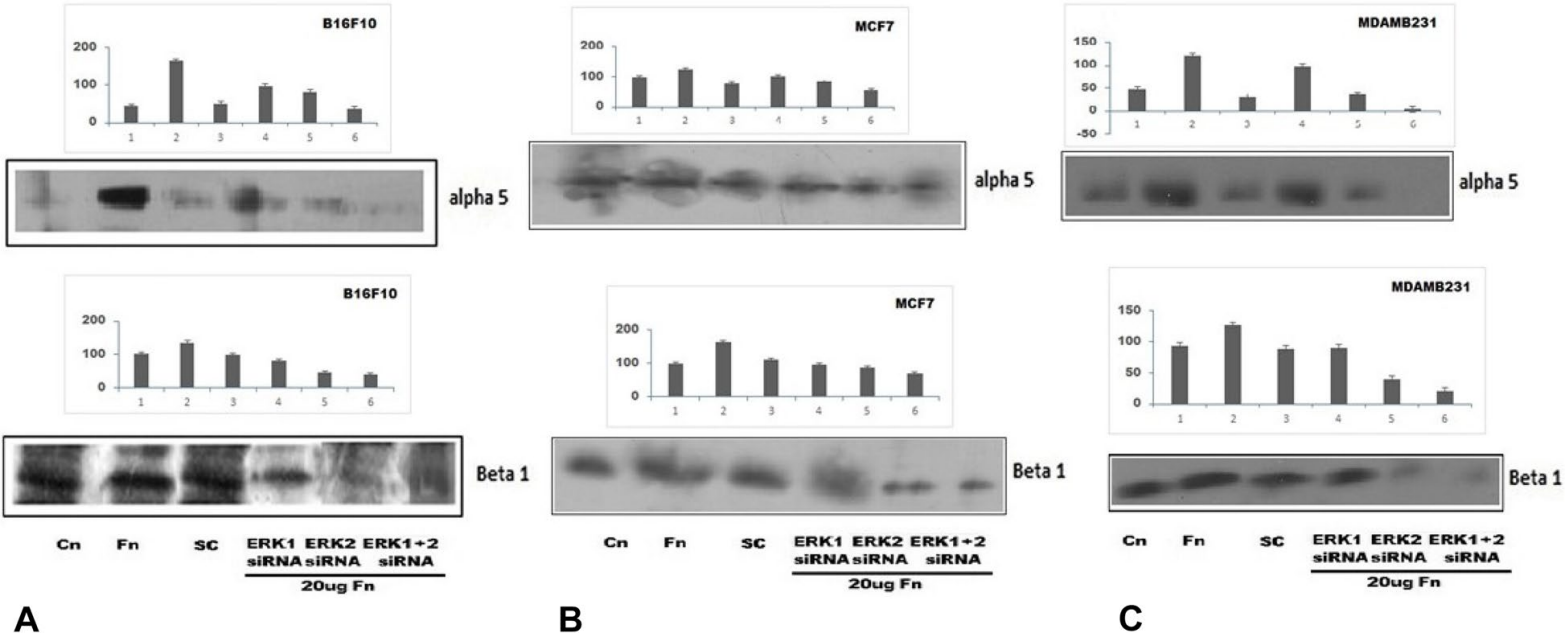
Effect of ERK1 and ERK2 inhibition on alpha-5 beta-1 integrin. For the transfection process, ERK1 siRNA, ERK 2 siRNA, ERK 1 + ERK 2 siRNA (sequential double transfection procedure), and negative control siRNA were transfected using Lipofectamine™ 2000 following standard protocol. Cells were then treated with 20-µg fibronectin (B16F10: 16 h, MCF7: 2 h, MDAMB231: 8 h), and the respective cell extract was subjected to Western blot by standard protocol. The immunoblot was probed for anti-alpha 5 and anti-beta 1 antibody. The blots representing B16F10 (**A**), MCF7 (**B**), and MDAMB231 (**C**) cells were developed using respective horse redox peroxidase (HRP) coupled second antibodies. The color was developed using West Femto as substrate. *β*-tubulin was used as loading control. The accompanying graph represents the comparative densitometric/quantitative analysis of the band intensities using ImageJ launcher (version 1.4.3.67) and arranged in the similar order as of the lanes. Data are means ± SEM of three experiments

The effect on integrin alpha-5 beta 1 after ERK1 and/or ERK2 inhibition is shown in Fig. 6. In Fig. 6A, alpha-5 levels increased appreciably after Fn treatment which decreased to basal levels when combinatorial siRNA transfections were done with ERK1 and ERK2 siRNA. ERK1siRNA treatment resulted in a 50% reduction of alpha-5 band intensity, while ERK2 siRNA further lowered it down. The beta-1 levels varied noticeably when ERK2 siRNA was introduced separately or in combination with ERK1 siRNA. Even the introduction of ERK1 siRNA along with fibronectin had a 40% drop down of band intensity with respect to Fn-treated and -untreated cells. The alpha-5 levels in MCF7 cells (Fig. 6B) had a slight alteration all through except when sequential double transfection of ERK1 and ERK2 siRNA was administered. Fn-treated cells had a marked increase of beta 1 which went down when ERK1siRNA and ERK2 siRNA were transfected separately. There was a drastic dropdown of beta-1 levels when combinatorial transfection was introduced. In Fig. 6C (MDAMB231 cells), ERK1 siRNA clearly decreased alpha 5 which further got lowered with ERK2 siRNA separately and in combination with ERK1 siRNA. Similar pattern of diminished beta-1 intensities was observed when ERK2 siRNA was treated separately and/or in combination with ERK1 siRNA. The scramble control (SC) did not show any difference with respect to control.

**Fig. 7:**
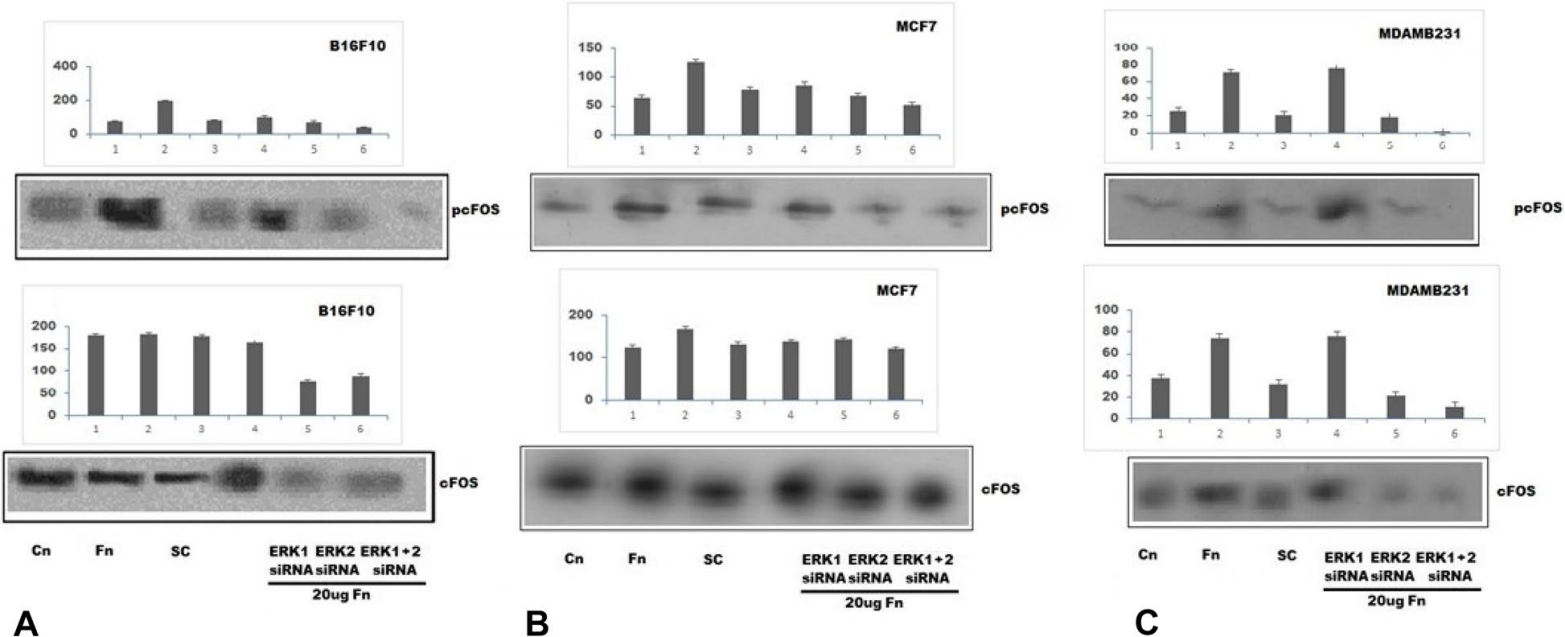
Effect of ERK 1 and ERK 2 inhibition on activity and expression of cFos. For the transfection process, ERK1 siRNA, ERK 2 siRNA, ERK 1 + ERK 2 siRNA (sequential double transfection procedure), and negative control siRNA were transfected using Lipofectamine™ 2000 following standard protocol. Cells were then treated with 20-µg fibronectin (B16F10: 16 h, MCF7: 2 h, MDAMB231: 8 h), and the respective serum-free medium was subjected to Western blot by standard protocol. The immunoblot were probed for anti-phospho cFos/anti-cFos antibody. The blots representing B16F10 (**A**), MCF7 (**B**), and MDAMB231 (**C**) cells were developed using respective horse redox peroxidase (HRP) coupled second antibodies. The color was developed using West Femto as substrate. *β*-tubulin was used as loading control. The accompanying graph represents the comparative densitometric/quantitative analysis of the band intensities using ImageJ launcher (version 1.4.3.67) and arranged in the similar order as of the lanes. Data are means ± SEM of three experiments

Figure 7 depicts the expression and activity of cFos after ERK1 and ERK2 inhibition. In Fig. 7A, there was a marked increase in the activity of phosphorylated cFos with Fn treatment. ERK1siRNA treatment lowers the band intensity of pcFos which further gets down with administration of ERK2 siRNA separately and in combination with ERK1 siRNA. The cFos expression did not change with Fn treatment and/or with ERK1 siRNA treatment. The expression altered considerably with ERK2 siRNA treatment both singly and in combination. The MCF 7 cells (Fig. 7B) showed similar levels of pcFos until ERK2siRNA was administered separately and/or in combination. There was no appreciable change in the expression of cFos total protein. Figure 7C showed the effect of ERK inhibition in MDAMB231 cells. There was no striking difference in pcFos when ERK1 siRNA was administered, though with introduction of ERK2 siRNA (separately and in combination) pcFos went down to basal levels. The trend was similar in cFOS expression.

**Fig. 8:**
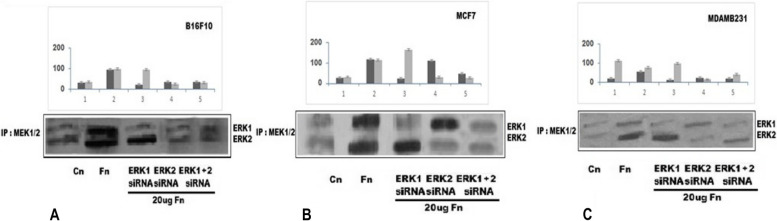
Interaction between MEK1/2 and ERK1/2. For the transfection process, ERK1 siRNA, ERK 2 siRNA, ERK 1 + ERK 2 siRNA (sequential double transfection procedure), and negative control siRNA were transfected using Lipofectamine™ 2000 following standard protocol. Cells were then treated with 20-µg fibronectin (B16F10: 16 h, MCF7: 2 h, MDAMB231: 8 h), and the respective serum-free medium was subjected to lysis by standard protocol. After preclearing for nonspecific binding, lysates were incubated with anti-MEK1/2 antibody overnight. Immune complexes were then incubated with Sepharose B beads, separated and resolved in SDS PAGE, and western blotted. The immunoblot were probed for anti-phospho ERK1/2 and anti-ERK1/2 antibody. The blots representing B16F10 (**A**), MCF7 (**B**), and MDAMB231 (**C**) cells were developed using respective horse redox peroxidase (HRP) coupled second antibodies. The color was developed using West Femto as substrate. *β*-tubulin was used as loading control. The accompanying graph represents the comparative densitometric/quantitative analysis of the band intensities using ImageJ launcher (version 1.4.3.67) and arranged in the similar order as of the lanes. Data are means ± SEM of three experiments

Figure 8 represents the interaction of MEK1/2 with ERK1/2 via co-immunoprecipitation assay. In B16F10 cells (Fig. 8A) following fibronectin treatment, there was a marked increase in the ERK1 and ERK2 levels. The ERK1 levels went down considerably with ERK1 siRNA usage, but the ERK 2 levels went up. With administration of ERK2 siRNA singly and in combination with ERK1 siRNA, the levels of ERK1 and ERK2 decreased to basal intensities. This pattern of ERK 1 and ERK 2 levels were similar in MDAMB231 (Fig. 8B), and introduction of ERK2 siRNA decreased band intensities of ERK1 and ERK2. In MCF 7 cells (Fig. 8C), the ERK1 levels went down considerably with ERK1 siRNA usage, but the ERK 2 levels went up and vice versa with respect to control. The combinatorial transfection reduced the expression of both the isomers to basal levels.

## Discussion

In B16F10 cells, pERK1 got activated with Fn showing its resemblance with pERK2. There was a 50% knockdown with ERK1 siRNA and the near-complete depletion when the knockdown was combined with ERK1 siRNA showing the specificity of the siRNA used. But surprisingly when ERK2 was knockdown, there was a rise in pERK1 levels suggesting pERK1 may compensate for ERK2 deficiency. The result was in accordance with the recent works done by Gagliardi et al. [15, 16]. The similar effect with ERK1 knockdown may imply a dependency between the isoforms. The immunoblots showing the levels of ERK1 and ERK2 suggested that in B16F10 cells, the results were more governed in the active protein level via phosphorylation. In MCF7, there was clear predominance of ERK2 level. An analysis performed in a recent paper showed that according to stoichiometry, ERK1 is four times less abundant than ERK2; even activated ERK1 is also four times less abundant than activated ERK2 in stimulated cells [17, 18]. Busca et al. showed that ERK 1/2 shows functional redundancy when works in an interchangeable state [19]. Fremin et al. confirmed that the development of embryo and placenta in mice having different combinatorial ratio of alleles of ERK1 and ERK2 is firmly linked with total ERK1/2 activity [20]. In MCF7 cells, ERK1 knockdown compensated the near-complete depletion of combinatorial knockdown of ERK2, both in activity and expression. This result may suggest that the predominant isoform was somehow dependent on the less abundant one for cellular expression. The striking rise in Fn-activated ERK1 and ERK2 may confirm the similarity in stimulating ERK1 as well as ERK2. In our result, the interdependency of ERK1 and ERK2 with respect to compensating each other was quite evident in MDAMB231 cells where the phospho form of both the proteins got elevated when each one was knockdown separately.

MMPs can degrade extracellular matrix and are implicated in progression and invasion in cancer. Elevated expression of MMP9 and MMP2 is associated with increased metastatic potential in many cancer types [15, 21–23]. To analyze the distinguishable role of ERK1 and ERK2 in controlling matrixmetalloproteinase activity, the three chosen cell lines were subjected to gelatin zymography. In B16F10 cells, separate knockdown of ERK1 and ERK2 could not fully reduce the activity of MMP9 showing reciprocity effect of each isoform (when the other one is unavailable). This was further implied when MMP9 got reduced to basal level when both ERK1 and ERK2 were hit down. Wang et al. found that in treated astrocytes, MMP9 level is reduced due to knockdown of ERK1 and ERK2 [24]. MCF7 cells which secret both MMP9 and MMP2 showed a differential effect on the protein knockdown. While MMP2 was reduced to basal level when ERK1 and/or ERK2 siRNA was introduced, MMP9 only got appreciably reduced when ERK2 was knockdown (separately and in combination). The higher levels of MMP9 with introduction of ERK1 siRNA suggest that ERK2 might have become more active in the absence of ERK1. The MMP9 activity in MDAMB231 cells might imply the dominance of ERK2 over ERK1, although depletion of ERK1 through siRNA clearly showed more than 50% knockdown in MMP9 levels. This lies in accordance with the interdependency of the two isoforms. The near depletion of MMP9 levels in all three cell lines might possibly propose that both ERK1 and ERK2 have substantial role in modulating MMP9 activity and subsequently cell invasiveness. The expression of MMP9 and MMP2 was also checked by Western blots after knockdown but did not show much difference throughout the lanes suggesting the total protein level remains unaltered (results not shown).

MEK1 and MEK2 are the only upstream molecules that can activate ERK1 and ERK2, and even MEK inhibitors are used to modulate ERK activity and subsequently cancer progression in clinical trials [25]. To understand the effect of ERK1 and ERK2 knockdown on the upstream molecules MEK1 and MEK2, we investigated the levels of the activating proteins by the immunoblots. In B16F10 and MDAMB231 cells, both the activity and expression of MEK1/2 remained intact throughout knockdowns of ERK1 and ERK2 (separately and in combination). In contrary, MCF7 cells showed an appreciable decrease in pMEK1/2 when sequential double transfection with ERK1 and ERK2 siRNA was administered suggesting a feedback regulation. To investigate phenotypical properties after inhibition of ERK1 and ERK2, the three cell lines were subjected to cell migration assay and cell invasion assay. In all three cell lines, cell migration assay and cell invasion assay showed a near complete inhibition of cell migration and invasion when both the protein isoforms were knocked down. This possibly implies the importance of the double inhibition uniformly. In B16F10 cells, the rate of cell migration was similar when ERK1 and ERK2 were inhibited separately suggesting the interdependency and the compensating effect of both the isoforms. Cavanaugh et al. showed that in MDAMB231, total ERK1/2 level was not altered when treated with trametinib, the MEK1/2 inhibitor [26]. In MCF7, ERK2 knockdown stopped considerably the rate of cell migration, whereas ERK1 knockdown increases the rate of cell migration suggesting ERK1 depletion might positively regulate cell migration. This was in accordance with recent findings and also with our results in the inability of controlling MMP9 activation via ERK1 siRNA. MMP9 might have played a role in cell migration during the knockdown of ERK1. In MDAMB231 cells, this effect was similar also suggesting the role of ERK2 in compensating ERK1. In cell invasion assay, B16F10 cells and MDAMB231 cells showed a similar level of cells invaded when either ERK1 or ERK2 was inhibited suggesting a strong dependency between the two. In MCF7 cells, inhibition of ERK1 contributed to the cell invasion, while inhibition of ERK2 restricted appreciably the rate of cell invasion suggesting the predominance and abundance of ERK2 in the cell.

There are many evidences, which suggest that integrins, which play a central role in cancer, cell anchorage differentiation, and migration, also regulate the production of MMPs among which the Integrin α5 and Integrin β1 have been shown to modulate release of MMP2 and MMP9 in various cancer cells. Moreover, there have been recent evidences, which suggest that ERK1/ERK2 associates with integrin [27, 28]. We analyzed the expression of integrins after ERK1 and ERK2 inhibition, and the results seem thought-provoking. There was a 50% reduction in both α5 integrin and β1 integrin levels in B16F10 cells suggesting that ERK1 abolition might have an effect on the integrins. The levels went further down with ERK2 inhibition and with combinatorial transfection implying an interrelation between the levels of two isoforms and the integrins. In MDAMB231 cells, the results were similar which might suggest a distinguishable role of ERK1 apart from ERK2 and also might imply the interdependency between the two isoforms in cancer cell signaling. The levels of α5 integrin and β1 integrin were more or less constant throughout siRNA treatments (single and combinatorial) though the levels went down appreciably with respect to the MCF7 cells treated with fibronectin alone. In order to investigate the mechanism behind the effector-function relationship downstream of ERK1/2, cFos levels were ascertained in these three cell lines. The pcFos expression went down in B16F10 cells with treatment of ERK1 siRNA and further depleted markedly with ERK2 siRNA showing the effect of ERK protein knockdown downstream of the molecule. There was also a depletion of cFos expression following ERK2 siRNA inhibition (single and in combination) supporting our hypothesis of distinguishable role of ERK2 as well as ERK1. In MCF7, the phospho levels of cFos went down considerably with siRNA treatment (ERK1 and ERK2: single and in combination) though the levels of dephospho-protein remained constant giving us a hint that there can be other signaling molecules (like JNK), which might play a role in modulating extra cellular matrix molecules along with ERK1/2. The results of MDAMB231 cells were showed no/less effect on cFos on ERK1 inhibition and more predominance of ERK2 (singly and in combination with ERK1) to regulate cFos activity and expression. Recent evidences suggest that stimulus-dependent activation of ERK1 and ERK2, which is true for many cancer models, depends on a competition between ERK1 and ERK2 in binding to the upstream kinase and their only activator MEK1/2 [29–31]. Hence, we wanted to see in our Fn-induced model the levels of ERK1 and ERK2 in MEK-ERK complexes. The immunoprecipitation studies showed that ERK1 inhibition might have prompted ERK2 to bind at higher levels with MEK2 to compensate the loss of the other isoform. In MCF7 in the absence of ERK2, binding of ERK1 to MEK appeared significantly increased implying the distinct role of ERK1 in compensating loss of ERK2. The results show that the abundance of ERK1 with respect to ERK2 holds a significance in interpretation of the nature of cell invasion and migration. As suggested by recent work, the cytosolic distribution of ERK1/2 is maintained by constitutive association with MEK1/2 proteins, which might hold true in our system resulting in the differential effect of ERK1 and ERK2 in cell invasiveness [32, 33].

## Conclusion

Research on cell signaling with respect to MAPK pathway and ERK has mainly focused on both the isoforms of ERK as a whole without much investigation on the individual role of ERK1 vis-à-vis ERK2. The present study tries to focus on the solitary yet distinct role of ERK1 and ERK2 and also caters the probability of their interdependency. The overall results if interpreted not only portray the contribution of ERK2 in compensating the loss of ERK1 and vice versa but also establish a clear role of ERK1 in cell invasiveness in fibronectin-induced system. More studies based on the transcriptional and nuclear activity of ERK1 and ERK2 should be promoted in order to get a complete idea of the complexity of their interdependence, which were beyond our present work. Proper interpretation of individual role and interdependency of ERK1 and ERK2 in a more comprehensive biochemical and biophysical model might help cancer prognosis and also assist in developing more targeted and successful small molecule inhibitors for cancer treatment.

## Data Availability

The authors confirm that the data supporting the findings of this study are available within this article.

## Abbreviations

MAPK: Mitogen-activated protein kinase
ERK: Extracellular signal-regulated kinase
MMP: Matrix metalloproteinases
Fn: Fibronectin
SFCM: Serum-free complete medium
